# The Utilization of Protective Face Masks among Polish Healthcare Workers during COVID-19 Pandemic: Do We Pass the Exam?

**DOI:** 10.3390/ijerph18020841

**Published:** 2021-01-19

**Authors:** Radomir Reszke, Łukasz Matusiak, Piotr K. Krajewski, Marta Szepietowska, Rafał Białynicki-Birula, Jacek C. Szepietowski

**Affiliations:** Department of Dermatology, Venereology and Allergology, Wroclaw Medical University, 50-367 Wroclaw, Poland; radomir.reszke@umed.wroc.pl (R.R.); luke71@interia.pl (Ł.M.); piokrzykrajewski@gmail.com (P.K.K.); marta.szepietowska0703@gmail.com (M.S.); rafal.bialynicki-birula@umed.wroc.pl (R.B.-B.)

**Keywords:** face masks, COVID-19, World Health Organization, healthcare workers

## Abstract

Relevant personal protective measures during the COVID-19 pandemic include face masks, possibly decreasing the risk of infection among the general population and healthcare workers (HCW) if utilized properly. The aim of the study was to assess whether different Polish HCW utilize face masks according to the 2020 World Health Organization guidance (WHO) criteria. This cross-sectional study included 1156 respondents who participated in an internet survey evaluating mask-related behaviors. All the WHO criteria were complied with by 1.4% of participants, regardless of medical profession, specialty or place of employment. HCW mostly adhered to criterion 1 (C1; strict covering of the face and mouth with the mask; 90.8%), C4 (washing/disinfecting the hands after touching/taking off the mask; 49%) and C3 (taking off the mask properly without touching the anterior surface; 43.4%), whereas C2 (avoidance of touching the mask with hands) was complied with least commonly (6.8%). HCW with mask-induced itch (31.6%) complied to C2 less often (odds ratio 0.53; *p* = 0.01). The study reveals that Polish HCW rarely adhere to all the 2020 WHO guidance criteria on the use of masks, whereas the adherence to particular criteria is variable and may be associated with the presence of skin-related conditions and other factors. Better compliance with the recommendations in the future is necessary to increase personal safety of HCW and prevent the transmission of SARS-CoV-2.

## 1. Introduction

The ongoing COVID-19 pandemic put to the test healthcare systems on all continents. As of 21 December 2020, there were over 75 million cases confirmed worldwide since the beginning of the pandemic [1], with 1.2 million in Poland. The alarming situation is further manifested with the number of officially reported deaths associated with COVID-19, with values exceeding 1.6 million worldwide and 26,000 in Poland. The official and obligatory safety regulations introduced by the Polish government necessitate social distancing (1.5 m between individuals) and covering of the mouth and nose in public spaces [2]. The latter includes streets, public transport, shopping facilities, workplaces and medical settings, to name just a few. Notably, when entering a public building (especially a healthcare facility), it is mandatory to disinfect the hands. With the continuous and increasing demand for the vaccination against SARS-CoV-2 to be finally made available to the public [3], it seems that strict adherence to recommendations concerning personal protective measures is still of crucial importance. Owing to the primarily airborne transmission of SARS-CoV-2 [4], masks and respirators are considered as relevant prophylactic factors against the spread of COVID-19 between humans, along with compliance with hand hygiene, physical distancing and other infection and control measures, as emphasized by the World Health Organization (WHO) and European Centre for Disease Prevention and Control (ECDC) [5,6]. With the progression of the pandemic, more data supports the use of face masks as a prophylaxis against SARS-CoV-2 transmission in the general public [7]. Personal protective measures remain valid especially in the context of healthcare workers (HCW) who are at an elevated risk of contracting COVID-19 when compared with the general population [8]. The transmission of SARS-CoV-2 to HCW may occur not only from infected patients but also in a community setting or between co-workers [9]. The rationale behind incorporating protective measures cannot be achieved if they are instigated improperly, possibly even promoting transmission of SARS-CoV-2 [5]. The reported compliance with the recommended safety measures during the COVID-19 pandemic is associated not only with an awareness of those measures but also with a psychological concept of self-efficacy both in HCW and in general public [10,11]. Concerning the utilization of face masks, previous studies have revealed that the lack of compliance with the safety guidelines is not uncommon in HCW and was reported both in pre-pandemic and current times [12,13]. The possible reasons encompass insufficient knowledge or conscious disregard of the recommendations, the lack of supplies due to rapidly increased demand [14] and certain inconveniences such as difficulties in breathing, warming or sweating, misting of the glasses, slurred speech or itching [15,16]. Unfortunately, the effective application of face masks may be compromised at multiple stages, beginning with the initial hygiene procedures of the hands, improper donning, redundant touching of the mask or the mask removal process. Moreover, further inadequacies include prolonged use of single-use masks, not replacing masks sufficiently often, reusing single-use masks or the lack of mask disposal [5].

Despite extensive medical knowledge and experience, strict and continuous adherence to all the safety guidelines may be problematic for HCW, especially in the developing COVID-19 situation. Therefore, accounting for the plethora of possible mistakes during seemingly obvious and repetitive procedures, our goal was to investigate whether Polish medical professionals do comply with the international guidelines regarding the correct use of face masks in everyday inpatient and outpatient settings.

## 2. Materials and Methods 

The cross-sectional study was based upon an original online survey created in Google^©^ Forms and then distributed to a wide range of HCW (physicians, nurses, supportive medical workers) working in inpatient and outpatient settings in Poland. The participation in the study was voluntary and the invitation was sent to HCW individually via WhatsApp^©^. According to the snowball sampling technique, each participant was also able to further send the invitation to their HCW contacts [17]. Besides demographics, the data on professional status, self-reported sensitive skin, atopic predisposition, current presence of a facial dermatosis and complaints of face mask-induced itch were also assessed. The utilized questionnaire is provided in Supplementary Material. The survey also included 7 questions based on the World Health Organization WHO guidance on the correct use of face masks as reported by Machida et al. [18] (Table 1). Each question could have been answered with “always/nearly always”, “often”, “rarely” or “never”. Compliance with a criterion was noted if the chosen answer was “always/nearly always”.

We purposefully omitted the question concerning hand hygiene and disinfection procedures prior to putting on the face mask (originally mentioned in the WHO guidance) [5] as this could have caused bias in our results. Currently, Polish regulations state that strict covering of the mouth and nose is mandatory while walking the streets, using public transport and while at work. Therefore, all HCW enter their workplace already wearing face masks. The data were collected between October 1st and 7th, 2020. We obtained data from 1156 respondents. Females constituted the majority of respondents (81,7%). The mean age of the subjects was 40.5 ± 11.8 years (range: 21–73 years). Based on the number of HCW in Poland [19], our sample size provided the confidence level of 99.9% and the real value was within ± 5% of the measured value. For statistical purposes, we performed Chi-square test and logistic regression analysis of the qualitative data where appropriate using Statistica 13 (Dell, Inc., Tulsa, OK, USA) software. The p-values below 0.05 were considered as statistically significant. The study was executed based on the statutory activity of the department, in accordance with the previously obtained approval of the Institutional Review Board.

## 3. Results

### 3.1. Basic Results

Our study participants declared their mean medical experience as 15.0 ± 11.8 years (range: 0–48 years). Among 1156 respondents, 60.6% stated their primary workplace as hospital, whereas outpatient settings concerned 39.4%. Physicians constituted the main respondents in our study (75.9%), outnumbering nurses (18.5%) and miscellaneous medical personnel (5.6%). The majority of HCW (41.4%) worked in internal diseases wards, followed by outpatient departments (39.4%) and surgery wards (13.4%), with the remaining group consisting of anesthesiology and infectious diseases ward employers. There was an equal division regarding positive or negative sensitive skin status (50.7% vs. 49.3%, respectively); over one third of the respondents (37.9%) confirmed personal or family atopic predisposition, whereas a similar percentage (37.4%) declared current presence of facial dermatosis (e.g., acne, seborrheic dermatitis, atopic dermatitis). The presence of itching during the previous week was reported by 31.6% of HCW.

### 3.2. Correct Use of Face Masks: The Entire Population of Respondents

Among the entire cohort of respondents, the compliance with all the criteria occurred rarely (1.4%). Regarding particular criteria, C1 (strict covering of the nose and mouth with the face mask), C4 (washing/disinfecting hands after face mask removal or touching) and C3 (taking off the mask properly without touching the anterior surface) were complied with most commonly (90.8%, 49%, 43.4%, respectively). On the other hand, the worst compliance mostly concerned C2 (avoidance of touching the mask with hands) (6.8%), followed by C6 (not reusing single-use masks) (33.7%) and C7 (disposal of single-use face masks after one use) (35.4%).

### 3.3. Correct Use of Face Masks: Different Medical Professions

Regardless of the exact medical profession practiced by HCW, adhering to all the criteria turned out to be very rare (1.1% in physicians, 2.8% in nurses, 0% in miscellaneous medical personnel), with no statistically significant differences (*p* = 0.11). A similar tendency was observed for C2 (7.2%, 6.5%, 3.1%, respectively). C1 was adhered to by the majority of HCW, irrespective of the position held in the medical facility (92.1%, 87.9%, 83.1%, respectively; *p* = 0.01), whereas all the other criteria were respected in less than half of the respondents in each group (Table 2). Statistically significant differences between the groups were observed concerning C7, C6, C4 and C1. Nurses were more likely to adhere to C7 (*p* < 0.001), C6 (*p* < 0.001) and C4 (*p* = 0.004) than physicians and miscellaneous medical personnel.

### 3.4. Correct Use of Face Masks: Inpatient vs. Outpatient Setting

HCW employed primarily in hospitals were most likely to adhere to C1, C4, C3 (89.4%, 46.9%, 41%, respectively), with a similar order presented by outpatient employees (93%, 52.4%, 47.1%). Both subgroups uniformly proved that complying with all of the criteria (1.1% vs. 1.8%, respectively) and to C2 (5.4% vs. 9%, respectively) poses major problems. It must be emphasized that outpatient workers declared themselves as more meticulous in adhering to the recommendations than hospital workers; the differences were statistically significant in the majority of the criteria (C7, C6, C2, C3, C1) (Table 3).

### 3.5. Correct Use of Face Masks: Different Medical Field of Specialty

Irrespective of the medical specialty, fulfillment of all the criteria was reported scarcely (0.6% in internal diseases ward, 1.3% in surgery ward, 4.5% among anesthesiologists and infectious disease specialists, 1.8% among outpatient workers) (Table 4). There were no statistically significant differences among the groups. C1 was adhered to by 90%, 91%, 81.8%, 93%, respectively (*p* = 0.02) and C4 by 46.8%, 43.9%, 54.5%, 52.4%, respectively. Statistically significant differences were observed concerning C6 (26.7%, 30.3%, 40.9%, 41%, respectively; *p* > 0.001), C7 (27.3%, 37.4%, 39.4%, 42.5%, respectively; *p* < 0.001) and C3 (39.5%, 40.6%, 53%, 47.1%, respectively; *p* = 0.03), with the lowest prevalence of adherence concerning internal diseases ward employees.

### 3.6. Correct Use of Face Masks: Skin-Related Conditions

All the criteria were fulfilled by 1.2–1.6% of HCW, notwithstanding the basic skin-related conditions (Table 5). C1 was complied with by the majority of HCW with sensitive skin (91.8%), atopic predisposition (91.3%), as well as those currently suffering from facial dermatosis (91.2%) and face mask-induced itch (89.6%). Each subgroup adhered to C4 in more than half of the respondents. The presence of face mask-induced itch favored the lack of compliance with C2 (odds ratio (OR) 0.53; *p* = 0.01). Conversely, these individuals were also more prone to adhere to C6 (OR 1.354; *p* = 0.01) and C7 (OR = 1.315; *p* = 0.02). Moreover, patients with sensitive skin were more likely to adhere to C3 (OR 1.394; *p* = 0.003), C4 (OR 1.508; *p* < 0.001), C6 (OR 1.471; *p* = 0.001) and C7 (OR 1.288; *p* = 0.02) (Table 6).

## 4. Discussion

According to WHO, face masks constitute an important component of the protective armamentarium against SARS-CoV-2 both in the general public and in HCW [5]. Recently, a Japanese study has evaluated the behaviors in over 2000 general public respondents in the context of the COVID-19 pandemic, with only 23.1% declaring adherence to all the WHO recommendations [18]. The correct use of face masks is mandatory to ensure their protective properties and it would seem that HCW should exceed the general population in this aspect. This is of utmost importance not only for limiting the spread of SARS-CoV-2, but also for maintaining discipline among HCW and to set an example for their patients [20]. However, the data in the literature revealed that HCW frequently experience problems in adhering to the guidelines on wearing face masks, even before the COVID-19 pandemic. As an example, in 2019 Herron et al. [12] reported that among 1034 surgically scrubbed HCW, only 18% fully adhered to the CDC guidelines on the correct use of face masks. The authors speculated that the improper use of face masks has contributed to surgical site infections over the years.

To the best of our knowledge, our study focused on the largest number of HCW evaluated specifically on the subject of face mask-associated behaviors during the COVID-19 pandemic to date. We clearly demonstrated that the adherence to all of the WHO recommendations concerning mask wearing was very poor among HCW, regardless of the profession, medical specialty or specific workplace. The novelty of our study stems from combining the significant HCW cohort of different professions and evaluating their behaviors according to the strict international guidelines on the utilization of face masks. Despite the definite value of the WHO recommendations, repetitive compliance with all of them each time is a challenge. Notably, HCW in our study adhered to all of the WHO recommendations less commonly than general public respondents (1.4% vs. 23.1%) as reported by Machida et al. [18]. However, the Japanese investigators adopted a slightly different way of assessing the adherence to the WHO recommendations, with both answers “always” and “sometimes” qualifying the respondent as compliant with a WHO criterion. Therefore, our method of assessment was more rigorous, as we strictly focused on HCW. Based on their professional knowledge and experience, as well as acknowledging their unique role in managing the developing COVID-19 pandemic, it seems reasonable to expect more strict adherence to the safety regulations, especially when compared with the general public. On the other hand, the authors of the previous study emphasized that Japan has a cultural habit of wearing face masks among the general public [18]. Nevertheless, our findings may still be regarded as alarming when taking into account the limitations of our study. Essentially, the subjective nature of self-reporting could be subject to recall bias among HCW, therefore the actual values might be even lower. Kumar et al. [13] reported that 88.2% of 392 Pakistani HCW demonstrated self-confidence regarding the knowledge on the correct use of face masks, yet when assessed concerning the procedural aspects, only 35.2% achieved good results. Recently, Supehia et al. [21] have reported that among 314 Indian HCW who used face masks during a 4-week period (as reported by external observers), 64.1% did it in a “correct manner”. The authors stated that they utilized a structured checklist based on the WHO guidance, although no specific data referring to particular WHO criteria was provided [21].

The participants of our study mostly adhered to meticulous covering of the nose and mouth (C1; 90.8% in total) as well as washing/disinfecting hands after face mask removal or touching (C4; 49%), and the tendency was constant among different subgroups. Interestingly, physicians seemed to adhere to C1 more commonly than nurses or miscellaneous medical personnel (*p* = 0.01). On the other hand, a higher proportion of nurses reported adherence to C4, as well as C6 (not reusing single-use masks) and C7 (disposal of single-use face masks after one use) (*p* = 0.004). The aforementioned observations are difficult to explain. HCW employed in outpatient settings were more likely to adhere to C1, C2, C3, C6, C7 (all p-values statistically significant) than those in inpatient settings. Possibly, the outpatient departments provide more time for HCW to follow the guidelines in each patient. Conversely, the higher number of patients approached each day also necessitates frequent repetition of the procedures. The varieties regarding compliance to C5, C6 and C7 may also stem from the periodic inadequacies of mask supplies or deficits in disinfectants (C4) [14,22]. This may partially account for the frequent non-compliance to all the WHO criteria in our study and prevent labeling HCW as purposefully neglectful of the standards. Among the conjoined group of anesthesiology and infectious disease department employees, we determined the lowest compliance with C1 when compared with other specialties (*p* = 0.02). The character of the procedures undertaken by these HCW (especially anesthesiologists) in the current context of COVID-19, requires significant mobility, frequently necessitating rapid actions, eventually impeding strict covering of the mouth and nose. Paradoxically, this observation may also be interpreted in a converse manner and seem surprising. Anesthesiologists are more exposed to patients possibly infected with SARS-CoV-2, especially due to procedures such as intubation. This requires close contact, increases the risk of contracting the infection and necessitates additional emphasis on personal safety.

Regardless of the different analyzed subgroups, over 90% of HCW consistently admitted that the avoidance of touching the mask with hands (C2) was not complied with. Moreover, HCW suffering from face mask-induced itch (31.6%) were less likely to comply with this criterion. The presence of itching creates the need to scratch which, even if not executed, might lead to repetitive mask touching and result in self-contamination [5,16]. Itching is not the only cause of mask touching, yet relieving this symptom should reduce the former behavior. As reported previously, individuals wearing face masks for longer periods of time (especially over 5 h) were more prone to experience itch, which occurred in 19.6% of the cohort (*n* = 1393) [16]. HCW in the present study wore face masks for longer periods of time due to their professional duties, therefore facilitating the occurrence of itch and eventually leading to mask touching. Other possible causes of the latter include discomfort associated with mask wearing, exacerbation of pre-existing acne, occupational dermatitis, seborrheic dermatitis or rosacea [23,24,25,26]. Nonetheless, certain studies demonstrated that wearing face masks tends to decrease face touching behaviors, both in the general public [27,28] and also in HCW [29]. Additionally, the self-reported prevalence of sensitive skin, acne and atopic predisposition in our cohort was similar to other epidemiological studies [30,31,32].

Despite a relatively simple nature and usefulness of the WHO guidance criteria these traits do not directly translate into their comprehensive and consequent application in medical settings. It is unknown if the situation is caused by the unawareness of those or if other factors play a role as well. Regardless, our findings clearly highlight the current need to perform educational campaigns aimed at HCW, including periodic training sessions on the correct utilization of masks. This may be further reinforced by infographics and video instructions provided online [33]. It could be of particular benefit to improve the overall promotion of these materials to reach a wider group of HCW, e.g., via social media.

Our cross-sectional study has several limitations, e.g., its online character, the point-prevalence and subjective nature of reporting by the respondents. Due to their profession, certain HCW may be hesitant to admit that they do not adhere to one or more safety criteria. Future studies on the topic should focus on a more objective assessment performed by external observers for a longer period of time. As older age seems to be associated with lower perceived stress during COVID-19 outbreak among the general public [34,35], it would be interesting to determine whether these factors affect the compliance with safety regulations among HCW in the future. Another disadvantage of our study stems from the chosen methodology which does not enable us to estimate the true value of the response rate. Still, this methodology is approved and utilized in various publications [36,37]. We did not determine the exact type of face masks utilized by our HCW. Thereby, certain respondents could have washed and disinfected them after single use, affecting the compliance with C6 and C7. Moreover, a relatively small group of anesthesiologists and infectious disease specialists participated in the study, despite being of particular importance in managing the COVID-19 pandemic. Therefore, statistical differences in face mask-wearing behaviors between different specialties must be interpreted with caution. Notably, our findings concern specifically Polish HCW and do not necessarily reflect the situation in other countries, especially outside Europe. Lastly, to the best of our knowledge, there are no data on mask wearing behaviors in Poland before the COVID-19 pandemic, both in the general public and among HCW. As a result, we cannot provide a comparison between the past and current situation.

## 5. Conclusions 

HCW do not pass the exam when it comes to strict compliance with all the WHO criteria on the safe use of face masks. However, rigorous and continuous compliance with all the criteria is and will remain a major challenge in everyday practice. The adherence to particular criteria is also variable, although it may be impaired by factors independent of HCW. Our study clearly implies that there is a continuous need for performing educational campaigns directed at HCW and expanding their social reach in order to achieve better compliance to the WHO guidance criteria. Acknowledging the shortcomings is a crucial step to improve the adherence to standards, thereby favoring safety in the workplace and possibly reducing the likelihood of a scenario in which HCW become the patients themselves.

## Figures and Tables

**Table 1:** The list of questions on the correct use of face masks according to the WHO guidance criteria.

Criterion	Question Content (as Translated from Polish)
C1	How often does the mask strictly cover your mouth and nose at work?
C2	How often do you happen to touch the mask at work?
C3	How often do you take the mask off properly at work (without touching its anterior surface)?
C4	How often do you clean/disinfect your hands after taking off/touching the mask at work?
C5	How often do you replace the mask when it dampens at work?
C6	How often do you reuse single-use masks?
C7	How often do you dispose of single-use masks after one use at work?

**Table 2:** Adherence to the WHO criteria among different medical professions.

Adherence to the WHO Criteria	All HCW (*n* = 1156)	Physicians (*n* = 877)	Nurses (*n* = 214)	Miscellaneous Medical Personnel (*n* = 65)	Chi-Square Value	*p*-Value
All criteria fulfilled	16 (1.4%)	10 (1.1%)	6 (2.8%)	0 (0%)	4.45	*p* = 0.11
C1	1050 (90.8%)	808 (92.1%)	188 (87.9%)	54 (83.1%)	8.76	*p* = 0.01
C2	79 (6.8%)	63 (7.2%)	14 (6.5%)	2 (3.1%)	1.64	*p* = 0.44
C3	502 (43.4%)	373 (42.5%)	103 (48.1%)	26 (40%)	2.52	*p* = 0.28
C4	567 (49%)	414 (47.2%)	126 (58.9%)	27 (41.5%)	10.93	*p* = 0.004
C5	444 (38.4%)	326 (37.2%)	87 (40.7%)	31 (47.7%)	3.39	*p* = 0.18
C6	389 (33.7%)	270 (30.8%)	97 (45.3%)	22 (33.8%)	16.29	*p* < 0.001
C7	409 (35.4%)	289 (33%)	100 (46.7%)	20 (30.8%)	14.92	*p* < 0.001

WHO—World Health Organization; HCW—healthcare workers.

**Table 3:** Adherence to the WHO criteria among inpatient and outpatient employees.

Adherence to the WHO Criteria	All HCW (*n* = 1156)	Inpatient Employees (*n* = 700)	Outpatient Employees (*n* = 456)	Chi-Square Value	*p*-Value
All criteria fulfilled	16 (1.4%)	8 (1.1%)	8 (1.8%)	0.76	*p* = 0.38
C1	1050 (90.8%)	626 (89.4%)	424 (93%)	4.19	*p* = 0.04
C2	79 (6.8%)	38 (5.4%)	41 (9%)	5.50	*p* = 0.02
C3	502 (43.4%)	287 (41%)	215 (47.1%)	4.25	*p* = 0.04
C4	567 (49%)	328 (46.9%)	239 (52.4%)	3.41	*p* = 0.06
C5	444 (38.4%)	261 (37.3%)	183 (40.1%)	0.95	*p* = 0.33
C6	389 (33.7%)	202 (28.9%)	187 (41%)	18.26	*p* < 0.001
C7	409 (35.4%)	215 (30.7%)	194 (42.5%)	16.90	*p* < 0.001

WHO—World Health Organization; HCW—healthcare workers.

**Table 4:** Adherence to the WHO criteria among HCW working in different medical departments.

Adherence to the WHO Criteria	All HCW (*n* = 1156)	Internal Diseases Ward Employees (*n* = 479)	Surgery Ward Employees (*n* = 155)	Anesthesiology and Infectious Diseases Ward Employees (*n* = 66)	Outpatient Employees (*n* = 456)	Chi-Square Value	*p*-Value
All criteria fulfilled	16 (1.4%)	3 (0.6%)	2 (1.3%)	3 (4,5%)	8 (1.8%)	6.97	*p* = 0.06
C1	1050 (90.8%)	431 (90%)	141 (91%)	54 (81.8%)	424 (93%)	9.39	*p* = 0.02
C2	79 (6.8%)	25 (5.2%)	9 (5.8%)	4 (6.1%)	41 (9%)	5.61	*p* = 0.13
C3	502 (43.4%)	189 (39.5%)	63 (40.6%)	35 (53%)	215 (47.1%)	8.61	*p* = 0.03
C4	567 (49%)	224 (46.8%)	68 (43.9%)	36 (54.5%)	239 (52.4%)	5.53	*p* = 0.14
C5	444 (38.4%)	168 (35.1%)	65 (41.9%)	28 (42.4%)	183 (40.1%)	4.10	*p* = 0.25
C6	389 (33.7%)	128 (26.7%)	47 (30.3%)	27 (40.9%)	187 (41%)	23.68	*p* < 0.001
C7	409 (35.4%)	131 (27.3%)	58 (37.4%)	26 (39.4%)	194 (42.5%)	24.50	*p* < 0.001

WHO—World Health Organization; HCW—Healthcare workers.

**Table 5:** Adherence to the WHO criteria according to the presence of skin-related conditions.

Adherence to the WHO Criteria	Sensitive Skin (*n* = 586)	Atopy (*n* = 438)	Pre-Existing Dermatosis (*n* = 433)	Face Mask-Induced Itch (*n* = 365)
All criteria fulfilled	7 (1.2%)	7 (1.6%)	6 (1.4%)	5 (1.4%)
C1	538 (91.8%)	400 (91.3%)	395 (91.2%)	327 (89.6%)
C2	43 (7.3%)	27 (6.2%)	27 (6.2%)	16 (4.4%)
C3	278 (47.4%)	205 (46.8%)	197 (45.5%)	155 (42.5%)
C4	317 (54.1%)	231 (52.7%)	221 (51%)	185 (50.7%)
C5	230 (39.2%)	171 (39%)	153 (35.3%)	138 (37.8%)
C6	222 (37.9%)	144 (32.9%)	152 (35.1%)	140 (38.4%)
C7	224 (38.2%)	158 (36.1%)	162 (37.4%)	145 (39.7%)

WHO—World Health Organization.

**Table 6:** Logistic regression parameters concerning the influence of skin-related conditions on the fulfilment of the WHO criteria as an effect.

Adherence to the WHO Criteria	Sensitive Skin (*n* = 586)	Atopy (*n* = 438)	Facial Dermatosis (*n* = 433)	Face Mask-Induced Itch (*n* = 365)
All criteria fulfilled	OR 0.754 95% CI (0.279–2.037) *p* = 0.29	OR 1.28 95% CI (0.473–3.460) *p* = 0.31	OR 1.00 95% CI (0.362–2.776) *p* = 0.004	OR 0.985 95% CI (0.340–2.855) *p* = 0.49
C1	OR 1.270 95% CI (0.850–1.896) *p* = 0.121	OR 1.101 95% CI (0.726–1.669) *p* = 0.32	OR 1.08 95% CI (0.712–1.636) *p* = 0.36	OR 0.809 95% CI (0.533–1.229) *p* = 0.16
C2	OR 1.175 95% CI (0.743–1.858) *p* = 0.69	OR 0.841 95% CI (0.52–1.361) *p* = 0.7	OR 0.858 95% CI (0.53–1.388) *p* = 0.27	OR 0.53 95% CI (0.302–0.931) *p* = 0.01
C3	OR 1.394 95% CI (1.104–1.761) *p* = 0.003	OR 1.247 95% CI (0.982–1.584) *p* = 0.04	OR 1.144 95% CI (0.9–1.454) *p* = 0.14	OR 0.944 95% CI (0.735–1.213) *p* = 0.33
C4	OR 1.508 95% CI (1.196–1.902) *p* < 0.001	OR 1.269 95% CI (1.0–1.61) *p* = 0.03	OR 1.136 95% CI (0.895–1.442) *p* = 0.15	OR 0.945 95% CI (0.738–1.212) *p* = 0.44
C5	OR 1.075 95% CI (0.848–1.362) *p* = 0.28	OR 1.044 95% CI (0.818–1.333) *p* = 0.36	OR 0.811 95% CI (0.634–1.038) *p* = 0.048	OR 0.964 95% CI (0.746–1.244) *p* = 0.39
C6	OR 1.471 95% CI (1.151–1.882) *p* = 0.001	OR 0.719 95% CI (0.543–0.951) *p* = 0.01	OR 1.109 95% CI (0.863–1.426) *p* = 0.209	OR 1.354 95% CI (1.045–1.755) *p* = 0.01
C7	OR 1.288 95% CI (1.011–1.064) *p* = 0.02	OR 1.050 95% CI (0.819–1.346) *p* = 0.35	OR 1.119 95% CI (0.874–1.434) *p* = 0.19	OR 1.315 95% CI (1.018–1.700) *p* = 0.02

WHO—World Health Organization.

## Data Availability

The data presented in this study are available on reasonable request from the corresponding author.

## Supplementary Materials

The following are available online at https://www.mdpi.com/1660-4601/18/2/841/s1, Document S1: The use of face masks during COVID-19 pandemic among healthcare workers.
