# Supplementary material for: The Utilization of Protective Face Masks among Polish Healthcare Workers during COVID-19 Pandemic: Do We Pass the Exam?
Source: Int J Environ Res Public Health. 2021 Jan 19;18(2):841. doi: 10.3390/ijerph18020841 (PMC7835780; doi:10.3390/ijerph18020841)
Supplement: Supplementary file 1 [file ijerph-18-00841-s001.pdf]

The use of face masks during COVID-19 pandemic among healthcare workers

1. **What is your primary workplace?**
    a. Internal medicine ward
    b. Surgery ward
    c. Anesthesiology or infectious diseases ward
    d. Outpatient department

2. **What is your profession?**
    a. Physician
    b. Nurse
    c. Miscellaneous medical personnel

3. **How many years have you been working in healthcare? ……………………..**

4. **Age: ……………………..**

5. **Sex:**
    a. Female
    b. Male

6. **Do you have a sensitive skin?**
    a. Yes
    b. No

7. **Do you have an individual or family predisposition to atopic diseases (history of atopic dermatitis, allergic rhinitis, allergic conjunctivitis etc.)?**
    a. Yes
    b. No

8. **Do you currently suffer from facial skin lesions (e.g. acne, seborrhoeic dermatitis, atopic dermatitis, etc.)?**
    a. Yes
    b. No

9. **Do you suffer from face mask-induced itch?**
    a. Yes
    b. No

10. [*If the answer to the question above is yes*] **Evaluate itch severity during the last week on a scale of 0-10 points.**

	0	1	2	3	4	5	6	7	8	9	10	
**No itch**	○	○	○	○	○	○	○	○	○	○	○	**The highest intensity you can imagine**

11. **How often does the mask strictly cover your mouth and nose at work?**
    a. Always/nearly always
    b. Sometimes
    c. Rarely
    d. Never

12. **How often do you happen to touch the mask at work?**
    a. Always/nearly always
    b. Sometimes
    c. Rarely
    d. Never

13. **How often do you take the mask off properly at work (without touching its anterior surface)?**
    a. Always/nearly always
    b. Sometimes

    c. Rarely
    d. Never

14. **How often do you clean/disinfect your hands after taking off/touching the mask at work?**
    a. Always/nearly always
    b. Sometimes
    c. Rarely
    d. Never

15. **How often do you replace the mask when it dampens at work?**
    a. Always/nearly always
    b. Sometimes
    c. Rarely
    d. Never

16. **How often do you reuse single-use masks?**
    a. Always/nearly always
    b. Sometimes
    c. Rarely
    d. Never

17. **How often do you dispose of single-use masks after one use at work?**
    a. Always/nearly always
    b. Sometimes
    c. Rarely
    d. Never
